# Congenital syphilis, still a reality in 21^st ^century: a case report

**DOI:** 10.1186/1752-1947-1-90

**Published:** 2007-09-19

**Authors:** Monica Chaudhary, Bineeta Kashyap, Preena Bhalla

**Affiliations:** 1 Department of Microbiology, Maulana Azad Medical College and associated Lok Nayak Hospital, New Delhi, India

## Abstract

Congenital syphilis is a preventable disease and its presence reflects a failure of prenatal care delivery systems, as well as syphilis control programmes. The procedure to prevent congenital syphilis through antenatal screening and treatment is well established. But implementation of effective programmes has proved very difficult especially in resource constrained countries.

## Background

Congenital syphilis is a rare and serious disease that although preventable continues to be a major health care problem [1]. Although the rate of congenital syphilis is declining in developed countries, a significant increase has been observed in the underdeveloped countries [2] in spite of the widespread use of penicillin to treat syphilis since the early 1950s. Late congenital syphilis (recognized 2 or more years after birth) [3] is a very rare clinical entity. We are reporting here a case of late congenital syphilis in order to emphasize that congenital syphilis still exists in 21^st ^century and global antenatal screening is mandatory to prevent this serious, yet largely preventable disease. We report here a case of late congenital syphilis who presented at the age of 13 years with palatal perforation.

## Case presentation

A 13 year old boy presented to the ENT OPD, at LN Hospital, New Delhi, India, with complaints of a hole in the hard palate which had been slowly enlarging, since its appearance 2–3 months back along with difficulty in eating and nasal speech.

**Figure 1:**
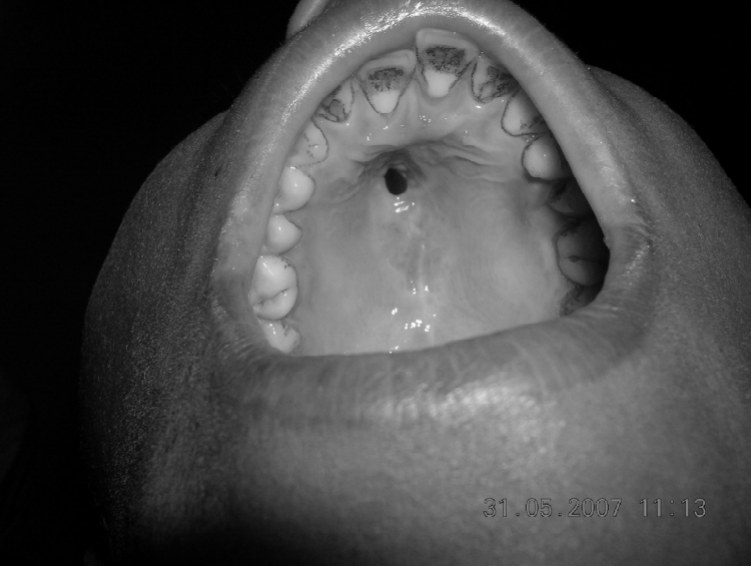
Perforation in the anterior hard palate.

Except for the presence of a perforation approximately 1 cm in diameter in the anterior hard palate, the physical examination of the child was otherwise unremarkable (Figure 1).

The routine investigations revealed Hemoglobin 12.4%, Total leukocyte count 11700/mm^3^, Differential leukocyte count – Polymorphs 57% Lymphocytes 30% Eosinophils 13%, and Platelet count 2.94 lac. Other routine biochemical investigations were insignificant. Venereal Disease Research Laboratory (VDRL) was reactive at 16 dilutions and *Treponema pallidum *haemagglutination test (TPHA) was positive (TPHA TEST KIT; Plasmatec Laboratory Products Ltd. UK). The X-ray of the chest was normal and Mantoux test was negative. Cerebrospinal fluid examination was normal. Skeletal survey revealed no radiological evidence of periosteal lesions or perichondritis. Ultrasound examination of the abdomen and pelvis showed no abnormality. No other orofacio-dental stigmata of congenital syphilis like Hutchinsons teeth were detected. Ophthalmic examination including fundoscopy was normal. No symptoms suggestive of nervous system involvement were observed. All the investigations were carried out before starting the treatment. Following these reports, the mother, father and three other younger siblings were investigated. The mother and the father were weakly reactive for VDRL and their TPHA was positive.

The younger siblings were all non reactive for VDRL as well as TPHA. All the family members including the patient were non reactive for HIV. The mother gave a history of taking some treatment for a genital lesion two years prior to the birth of the patient, The records of the above-mentioned condition or any treatment taken were not available. The obstetric history of the mother was uneventful. The patient was a product of unbooked full term normal vaginal delivery at home by traditional birth attendants. Our patient had three younger siblings who were also full term normal vaginal delivery and all are alive and well. Except for the development of some rash in the diaper area as a neonate there was no other history of characteristic bullous lesions elsewhere on the body. The developmental milestones of the patient were normal as told by the mother. The patient gave no history of any trauma, sexual assault or child abuse or drug abuse.

The child and his parents were started on a 3-week course of i/v aqueous crystalline Penicillin G 50,000 U/kg (at 8-hour intervals). He was informed of the risk of further enlargement of the defect and was asked to come back for palatal repair on completion of the medical treatment. He tolerated treatment well without any complications and showed a fall in VDRL titers to 2 dilutions when last seen after 6 months of his first visit.

## Conclusion

Congenital syphilis represents a significant financial and emotional burden in developing countries. Even one case of congenital syphilis is a sentinel public health event, since timely diagnosis and treatment of syphilis infected pregnant woman should prevent transmission almost entirely [4]. The risks of vertical transmission and fetal diseases are directly related to the stage of maternal syphilis during pregnancy. It is estimated that in women with syphilis of a few years duration, about half of the pregnancies will be affected, with one half of the affected pregnancies ending in stillbirth (including miscarriages), and the other half in perinatal death or serious neonatal infection (congenital syphilis) [5]. The risk of fetal loss and congenital syphilis drops slightly in early latent stage and decreases to 10% in late latent stage, respectively [6]. As seen in our case too, the patient was the eldest child of the couple, who was born 2 years after the detection of a genital lesion in the mother but the subsequent pregnancies were unaffected, signifying the fact that the risk of vertical transmission and fetal disease is directly related to the stage of maternal syphilis during pregnancy. More than 50% of live born affected infants may be asymptomatic and many of them are often not reported [7].

Differential diagnosis of a lesion presenting as palatal perforation should include tertiary syphilis, leprosy, tuberculosis, mucormycosis, mechanical trauma, intranasal cocaine abuse, malignancies, especially nasal T cell lymphomas, Wegener's granulumatosis, sarcoidosis and midline non-healing granuloma [8]. A positive VDRL and TPHA test along with a detailed family history helped us to arrive at the diagnosis of late congenital syphilis.

A recent data from WHO states that only 68% of women in developing countries receive antenatal care and of these about half do not attend ANC clinics until after the first trimester[9]. A study from Russia has stated that early diagnosis and appropriate treatment of syphilis infected mothers, which is the mainstay of congenital syphilis prevention, cannot occur when 60% of at-risk women do not access early prenatal care [4]. The late presentation of our case re-emphasizes the fact that antenatal services need to be strengthened.

It is difficult to discuss such a socially stigmatized disease, but if congenital syphilis is to be targeted for elimination, new approaches are required. The true incidence must be determined, diagnostic measures improved and risk factors controlled. Countries need to reexamine their current policies related to antenatal care and steps must be taken to overcome all administrative and cultural barriers. Control measures must be based on mandatory antenatal screening in 1^st ^trimester supported by treatment and partner notification with adequate follow up. ANC must be strengthened to ensure that there is no reinfection by treating all sexual partners, promoting condom use during pregnancy and counseling all women on how to prevent sexually transmitted infection.

A study from rural Haiti has shown that decentralizing screening for syphilis can drastically reduce the incidence of congenital syphilis even with limited infrastructure in peripheral areas [10]. With the availability of rapid, point-of-care diagnostic tests, even the lowest level of health care delivery system can benefit. The current widespread scale up of PMTCT (prevention of mother to child transmission) programme for HIV prevention in antenatal clinics offer a golden opportunity to promote early attendance and routine testing for syphilis and HIV. Resources used to develop appropriate infrastructure for either programme can benefit both HIV and syphilis prevention programmes. Only a substantial effort can make congenital syphilis a tragedy of past.

## Competing interests

The author(s) declare that they have no competing interests.

## Authors' contributions

MC carried out the detailed case study, collected references and review material, planned the study and drafted the manuscript. BK carried out the serological assays and participated in the design of study. PB conceived the study, and participated in its design, coordination and value addition and final approval of the manuscript. All authors read and approved the final manuscript.
